# Effects of Darkness and Light Spectra on Nutrients and Pigments in Radish, Soybean, Mung Bean and Pumpkin Sprouts

**DOI:** 10.3390/antiox9060558

**Published:** 2020-06-26

**Authors:** Linda Mastropasqua, Nunzio Dipierro, Costantino Paciolla

**Affiliations:** Department of Biology, University of Bari “Aldo Moro”, Via E. Orabona 4, 70125 Bari, Italy; linda.mastropasqua@uniba.it (L.M.); nunzio.dipierro@uniba.it (N.D.)

**Keywords:** sprouts, vitamin C, light treatments, antioxidants, phytonutrients

## Abstract

Fresh sprouts are an important source of antioxidant compounds and contain useful phytonutrients in the human diet. Many factors, such as the time of germination and types of light, influence the physiological processes and biosynthetic pathways in sprouts. The effect of red, blue and white light vs. dark conditions on the quality parameters in different sprout species after 5 d of germination was evaluated. Total ascorbate, soluble proteins, sugars, phenolic compounds, and pigments, such as carotenoids, chlorophylls, and anthocyanins, were investigated in radishes, soybeans, mung beans, and pumpkin sprouts. The light treatments increased the contents of vitamin C and the various pigments in all sprouts, conversely, they increased the soluble proteins and sugars, including d-glucose, d-fructose and sucrose, in soybeans and pumpkins, respectively. The dark treatment prevented the decrease in dry matter due to the lighting, while the red light induced an increase in polyphenols in soybean. These results suggest that the nutritional content of different sprouts grown under different light conditions depend on the dark or specific spectral wavelength used for their growth. The manuscript may increase the knowledge on light use for the industrialized food production aiming at preserving the phytonutrient content of vegetables, increasing the consumer health, or developing tailored diets for specific nutritional needs.

## 1. Introduction

In recent years, the use of sprouts, particularly in vegan and vegetarian diets, has become a topic of interest to model the “Mediterranean diet”. Careful consumers take advantage of introducing fresh plant foods into their diets, such as sprouts, that are rich in important nutrients, easy to digest and low cost. The sprouts originate through the germination of seeds in a process in which physical, chemical and metabolic changes occur and where proteins and polysaccharides are converted into amino acids and sugars. In some legumes, germination results in an increase and decrease in vitamins and anti-nutritional factors, respectively [1], such as an increase in antioxidant compounds [2] and angiotensin converting enzyme [3], which has been observed. Shah et al. [4] reported that the process of germination improved the nutritional value of mung beans in terms of a higher concentration of nutrients, reduced phytic acid, and improved protein content and ascorbic acid. In addition, the important functional properties of the sprout nutrients have attracted attention as potential health-promoting functional foods [5]. Several potential anti-hypertensive, anti-hyperlipidemic, or antidiabetic compounds have also been identified in germinating seeds [5,6]. The sprouts of mung beans and soybeans have been shown to be more effective for an anti-hypertensive diet [7]. The sprouts of cruciferous plants, such as radishes and broccoli, contain high levels of CoQ10, which is considered to be a potential anti-hypertensive and anti-hyperlipidemic compound [8].

The temperature, time of germination and light influence the biochemical and nutritional properties of the sprouts [9]. Significant differences in the polyphenol, flavonoid and phenolic acid content in legume seeds before and after germination in darkness at 30 and 40 °C for 5 d are reported [7]. The light induces many developmental and physiological responses in the life cycle of the plant, including germination. The wavelength, irradiance, photoperiod and direction of the light influence the growth of the plants. Red and blue light induce a greater increase in growth and development, as they increase the photosynthetic process and regulate various responses in higher plants [10,11,12]. The synthesis of vitamin C (l-ascorbic acid; AsA), an important antioxidant compound, is light dependent as observed in *Avena* [13], broccoli [14] and asparagus [15] post-harvest. In addition, blue light is efficient at inducing anthocyanin biosynthesis [16,17]. A diet rich in flavonoids, such as anthocyanins, decreases the rate of death from cardiovascular diseases, stimulates the responses of the immune system and reduces the inflammatory process. Dietary sprouts are generally grown in darkness and appear to be yellow in colour due to their chlorophyll deficiency and are utilized uncooked. When the sprouts are exposed to the light, the genes involved in the chlorophyll and carotenoid biosynthesis are upregulated with subsequent greening [18]. However, very little attention has been paid to the role that the light quality has on phytonutrients of dietary sprouts. Therefore, the aim of this work was to provide a more in-depth investigation of the effects of white (WL), blue (BL) and red (RL) lights, and darkness (D) on four types of sprouts usually utilized for food. In particular, qualitative parameters related to physiological and biochemical processes, such as soluble sugars (d-glucose, d-fructose and sucrose), starch, soluble proteins, chlorophyll a and b and carotenoids, and compounds related to human health, such as vitamin C, anthocyanins and total phenols, were investigated in radish, soybean, mung bean and pumpkin sprouts grown under different light treatments.

## 2. Materials and Methods

### 2.1. Plant Materials, Seed Germination and Light Treatments

Seeds of soybeans (*Glicine max* L. Merr), mung beans (*Vigna radiata* L. Wilczek), radishes (*Raphanus sativus* L.) and pumpkins (*Cucurbita moschata* Duch) were purchased from Bivacchi Spa (Perugia, Italy). The seeds were sanitized with 0.5% sodium hypochlorite solution (*v*/*v*) for 15 min and then thoroughly washed for 5 min with distilled water to remove any trace of sodium hypochlorite and soaked in distilled water for 2 h. Two batches of seeds of each plant species (10 g) were placed on two layers of filter paper (Whatman Grade 1) into Petri dishes (12 cm diameter). The filter paper was moistened with 5 mL of distilled water once a day. The Petri dishes were incubated in a plant growth chamber at 24 ± 1 °C with 78 ± 2% of relative air humidity under different light conditions with a 16 h photoperiod or in continuous darkness (D).

White light (WL) was provided by a T8 L18W/835 Osram 26Ø fluorescent tube with a wavelength range of 400–700 nm, and three different peaks: blue at 440 nm, green at 545 nm, yellow at 580 nm and red at 610 nm. Blue light (BL) was provided by a T8 L18W/67 Osram 26Ø fluorescent tube with a wavelength range of 400–460 nm and a peak at 440 nm. Red light (RL) was provided by a T8 L18W/60 Osram 26Ø fluorescent tube with a wavelength range of 600–630 nm and a peak at 610 nm. The photosynthetic photon flux density for each light source at the Petri dish surface were 110 ± 1 µmoL m^−2^ s^−1^ measured with a Photo-Radiometer, Model HD2302.0 (Delta OHM SRL, Pordenone, Italy) according to the manufacturer’s instructions.

**Figure 1:**
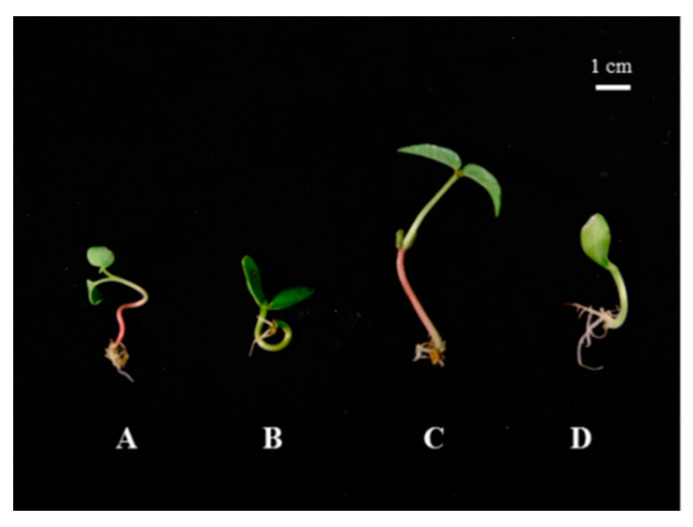
Sprouts of radishes (**A**), soybeans (**B**), mung beans (**C**) and pumpkins (**D**) grown in white lights (WL) 5 d after germination. The sprouts showed morphological differences. The soybean and mung bean sprouts in WL had fleshy cotyledons that were also present when the young leaves appeared, while in radishes and pumpkins, the cotyledons changed into photosynthetic leaflets.

Five days after germination, when reaching commercial length, the sprouts were collected, washed with sterile water, dried with absorbent paper and analysed. For instance, a picture of the shoots used is shown in Figure 1.

### 2.2. Determination of Dry Matter Content

The dry matter was determined in five-day sprouted seeds. For the analysis, lots of 30 random sprouts of each sample were weighed (fresh matter) as quickly as possible to limit the losses through evaporation and dried at 105 ± 2 °C in an oven until a constant weight was observed (approximately 24 h). The dry matter content was expressed as a percentage of the ratio of constant weight following drying and the initial fresh matter.

### 2.3. Anthocyanin Content

The anthocyanin content was determined as reported in [19]. Briefly, each sprout sample (5 g) was cut into pieces and incubated at 65 °C for 2 h with 20 mL of a solution containing 98% methanol and 0.24 M HCl. After centrifugation at 4500× *g* for 10 min, the anthocyanin content was measured spectrophotometrically at both 530 nm and 657 nm. The formula A_530_ − 0.25 × A_657_ that corrects the absorbance for the chlorophyll degradation product was utilized.

### 2.4. Determination of the Chlorophyll and Carotenoid Contents

The chlorophyll and carotenoid contents were determined as described by Lichtenthaler [20] with some modifications. Five grams of fresh material was homogenized with 10 mL of absolute acetone and 3 mg of Na_2_CO_3_ and centrifuged at 20,000× *g* for 15 min. The absorbance of the supernatant was measured spectrophotometrically at 645 and 662 nm, respectively, for chlorophyll a and b and at 470 nm for the carotenoids. The total chlorophyll and carotenoid content was calculated using Lichtenthaler’s equations.

### 2.5. Vitamin C Content

Five grams of fresh tissue was homogenized with ten volumes of cold 5% (*w*/*v*) metaphosphoric acid in a porcelain mortar. The homogenate was centrifuged for 15 min at 20,000× *g*, and the supernatant was collected for the analysis of l-ascorbic acid (AsA) and l-dehydroascorbic acid (DHA) as described in [21].

### 2.6. Soluble Protein Assay 

At various times, 5 g of sprouts was ground by pestle and mortar in 15 mL of extraction medium containing 100 mM potassium phosphate buffer pH 7.0, 0.5 M sorbitol, 1 mM EDTA and 0.05% (*w*/*v*) cysteine. After centrifugation (20,000× *g*, 20 min, 2 °C), the soluble fraction was desalted by dialysis against 50 mM Tris-HCl, pH 7.8. This desalted fraction was used to quantify the protein content with a Protein Assay kit from Bio-Rad (Hercules, CA, USA) with bovine serum albumin as the standard [22]. The reproducibility of Bio-Rad kit, expressed as coefficient of variation (%CV), is 2% approximately; the lower limit of detection for protein molecular weight is 3000 to 5000 daltons.

### 2.7. Soluble Carbohydrates and Starch Measurements

Five grams of sprouts from each treatment was cut into pieces and analysed. After hot extraction (80 °C) with ethanol (95%) and centrifugation at 4500× *g* for 10 min, the levels of mono- and di-saccharides (d-glucose, d-fructose and sucrose) were determined spectrophotometrically using a Megazyme kit (Sucrose/d-Fructose/d-Glucose Assay Kit; K-SUFRG, Megazyme, Ireland) according to the manufacturer’s protocol. The reproducibility of the K-SUFRG is for D-Glucose %CV = 0.76, for D-Fructose %CV = 0.87, for Sucrose %CV = 0.24.

The alcohol insoluble fraction (pellet) was used to determine the starch content using a Megazyme kit (Total Starch Assay Kit AA/AMG; K-TSTA, Megazyme, Ireland) according to the manufacturer’s protocol. Reproducibility value is %CV = 3%. The Total Starch Kit can accurately measure starch levels as low as 1% *w*/*w*.

### 2.8. Determination of the Polyphenol Contents

The fresh tissue was homogenized with a 30% ethanol solution in a 1:2 weight/volume ratio. The homogenate obtained was diluted at 1:6 with distilled water. The sample was incubated at 80 °C for 1 h in darkness and centrifuged at 7000× *g* for 10 min. For each sample, 0.3 mL of supernatant was utilized for analysis as described in [23]. The total phenolic content was determined according to a calibration curve of gallic acid. The results were expressed as mg of gallic acid equivalent (GAE) for 1 g of fresh matter.

### 2.9. Starch Qualitative Analysis

The seeds of soybeans and mung beans were soaked in distilled water for 2 h. Cotyledon transversal sections (15 µm thick) were cut with a DSK-1000 vibratome (Dosaka, Kyoto, Japan) and stained with Lugol solution (I_2_KI) diluted (Lugol:distilled water, 1:40) for 10 min. The sections were mounted on standard microscope slides and examined with a BX-40 light microscope Olympus and DP-21 digital camera (Olympus America Inc., Center Valley, Pennsylvania, PA, USA).

### 2.10. Statistical Analyses

The data reported are the average of at least three replicates from four independent experiments. A one-factor ANOVA was performed on the observed means of the compound content for each sample, and the significance of the different treatments (white, blue, and red light) within each sample with respect to the dark control was evaluated using Tukey’s HSD test for multiple comparisons (*p* < 0.05).

## 3. Results and Discussion

### 3.1. Effect of Light Spectral Properties on Dry Matter and Soluble Proteins

**Figure 2:**
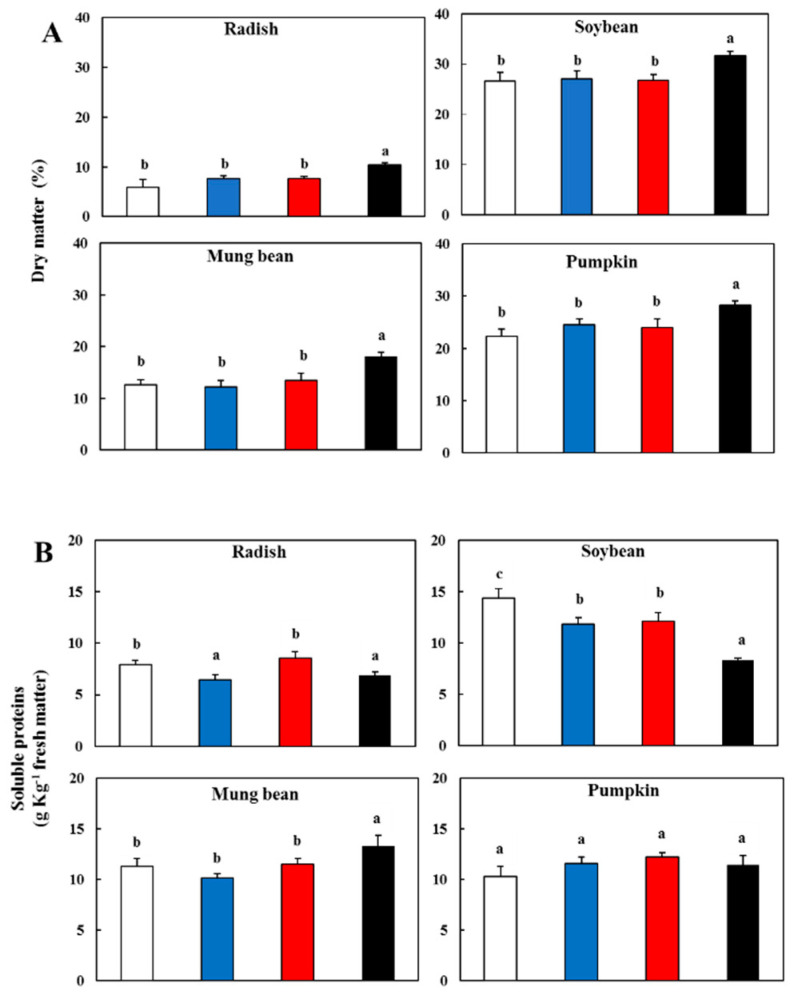
(**A**) Dry matter content in the sprouts 5 d after germination following various treatments. (**B**) Content of the soluble proteins in the sprouts 5 d after germination following various treatments. Values represent the means of at least three replicates from four independent experiments. Identical letters over the columns indicate non-significant differences between the treatments within each sprout (Tukey’s HSD test, *p* < 0.05). White light (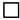); blue light, (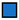); red light (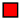), and dark (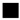).

Water was the primary component of the fresh matter in the sprout (represented by the apex, cotyledons, hypocotyl and root). However, differences in the dry matter occurred among the various sprouts that were treated differently. The highest value of dry matter was observed in those that were grown in darkness (Figure 2A).

The lighting caused a lower value in the dry matter than the darkness, and different types of light did not exhibit a significant difference. Soybeans showed the highest value in both the light and dark treatments followed by pumpkins, mung beans and radishes. The trend observed in all the sprouts, that there was more high dry matter in darkness and with the highest value in soybeans, was probably due to the abundant amount of storage compounds contained in the cotyledons of the shoot. Indeed, during the soybean germination, the storage compounds are mainly utilized in the sprouts grown in the light rather than in darkness. Blue, red and white light caused a larger utilization of storage with the subsequent loss of dry matter. This was probably due to a higher active metabolism under light conditions, as also reported by Chen and Chang [24], in which a significant loss of lipids occurred in soybean sprouts. On the other hand, the preservation in darkness of dry matter was probably due to a lower metabolic activity for the low consumption of sugars and starch as observed. However, other components, such as cell wall polysaccharides, lipids and mineral salts, may contribute to the dry matter.

In the dry soybean seeds, the proteins represent the prominent type of storage accumulated in the cotyledons. In the soybean sprouts, the content of the soluble proteins was higher than that of the other sprouts after light exposure (Figure 2B). The soluble protein content significantly increased (*p* < 0.05) in the soybean in all light treatments and in radish sprouts in the WL and RL compared to the dark. After five days of germination, the WL became the most effective, and the high presence of soluble proteins suggested the beginning of photosynthetic activity in the greening cotyledons of the soybean. No significant difference in the different light treatments with respect to the dark was observed in pumpkins, while there was a decrease in mung beans.

### 3.2. Effect of Light on Sugars and Starch

**Figure 3:**
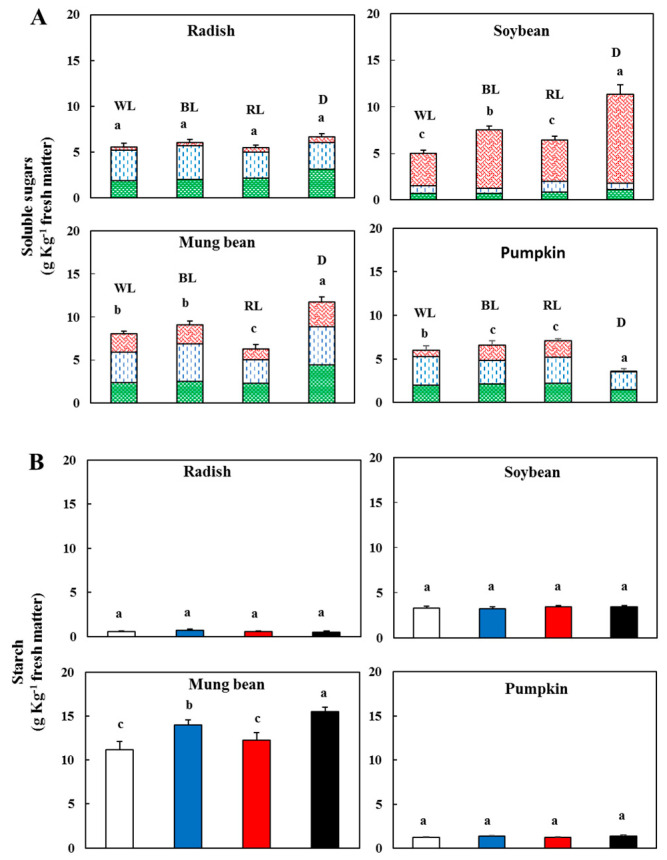
(**A**) Contents of soluble sugars—d-fructose, (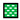), d-glucose (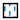), sucrose (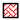)—In sprouts 5 d after germination following various treatments. White light, WL; blue light, BL; red light, RL, and dark, D. (**B**) Starch content in the sprouts 5 d after germination following various treatments. White light (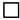); blue light (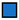); red light (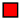), and dark (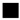). Values represent the means of at least three replications from four independent experiments. Identical letters over the columns indicate non-significant differences between the treatments within each segment (Tukey’s HSD test, *p* < 0.05).

Light spectrum properties strongly influence the physiology, growth, development and phytochemical accumulation in planta, particularly for vegetables produced in controlled environments [25]. The common sugars are phytochemicals classified as primary metabolites. Total sugar content (d-fructose plus d-glucose plus sucrose) of the different sprouts in both the dark and the different light conditions is shown in Figure 3A.

In darkness, the total sugar content was significantly (*p* < 0.05) higher in mung beans and soybeans; in particular, the value of the soybean was 55% lower when grown under WL than in darkness. In mung beans, the RL induced the 53% decrease in soluble sugar content with respect to the dark. In the soybean seeds, the lipids represent 22% of the dry matter [24]. During germination, the lipids are decomposed to succinate that in other cell compartments (mitochondria and cytosol) are converted in sugars, primarily sucrose. In soybeans, the di-saccharide sucrose was the more abundant sugar among those analysed, and it was 84% of the sum of the three sugars in darkness (Figure 3A).

Interestingly, since it is not a reducing sugar, the sucrose could be transported across distances and this might serve to allow for the growth and development of the soybean sprouts. The lipids are also catabolized in the dark, in which the gluconeogenesis process in dark conditions allows for the etiolated seedlings to grow and develop in the absence of photosynthesis. In the soybean seeds, the storage tissues are poor in starch and rich in lipids that are probably converted into sucrose during germination. After five days from germination, the persistence of the high sucrose content in both the dark and light conditions, highlights a surplus of sucrose, probably due to a limited or lack of utilization. In contrast, in radishes, mung beans and pumpkins, the di-saccharide sucrose was the 26% maximum of the total sugars, while both d-glucose and d-fructose are more abundant. In the pumpkin sprouts, the increase in soluble sugars in the light may be due in part to the high photosynthetic level for the presence of the great greening cotyledons. The significant decrease in the total sugar content in mung beans and soybeans could be correlated with higher consumption. Generally, the different content of the sugars in the various sprouts suggested that the responses of sprout to light qualities were related to species or cultivars as well as the growth period and light intensity as just reported in literature [26,27]. Particularly, the lower sucrose content in soybean sprouts in WL compared to the BL was probably caused by its consumption in cell growth processes with a possible involvement of phototropins. Indeed, in BL, the hypocotyl was less grown than in WL (data not shown) where sucrose was utilized for a greater growth of the sprout. It is reported that BL rapidly and strongly inhibits hypocotyl elongation [28], reduces the plant height [29] and the BL-activation of phototropin (nph1) influences cryptochrome signaling leading to growth inhibition [28]. However, further detailed studies, comprising also the activities of sucrose-metabolism associated enzymes, including sucrose synthase, sucrose phosphate synthase and invertases [30], are needed for a better comprehension of the effects of selected spectral light on sprouts qualities and are in progress.

Red light is needed for starch accumulation and for the proper development of the photosynthetic apparatus [31]. The starch content after the different light conditions and dark treatment is shown in Figure 3B. The highest content was observed in mung beans (15 in darkness and 14 mg g^−1^ of fresh matter in BL, respectively). Soybeans had the next highest content with a value of approximately 3 mg g^−1^ of fresh matter in all the treatments. Low levels and no significant difference under different lighting conditions and darkness were observed in pumpkins and radishes.

**Figure 4:**
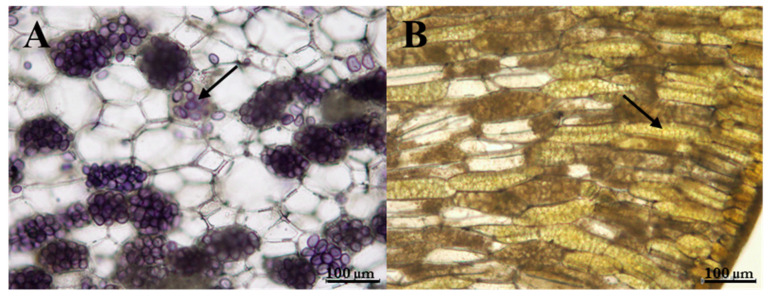
Light micrograph of transversal section of the mung bean cotyledon; arrow indicates the starch grains (Panel (**A**)). Light micrographs of transversal section of the soybean cotyledon; arrow indicates the protein vacuoles (Panel (**B**)).

Many starch grains were observed in transverse sections of the mung bean cotyledons (Figure 4, Panel A). In soybeans, although the cotyledons had been incubated with the reagent of Lugol for the same time utilized for mung beans, the material did not show the presence of starch grains, while numerous protein vacuoles were observed (Figure 4, Panel B). Interestingly, due to the low sugar and starch levels, the radishes and pumpkin sprouts could be utilized for low carbohydrate diets.

### 3.3. Changes in the Vitamin C and Total Phenolic Contents

**Figure 5:**
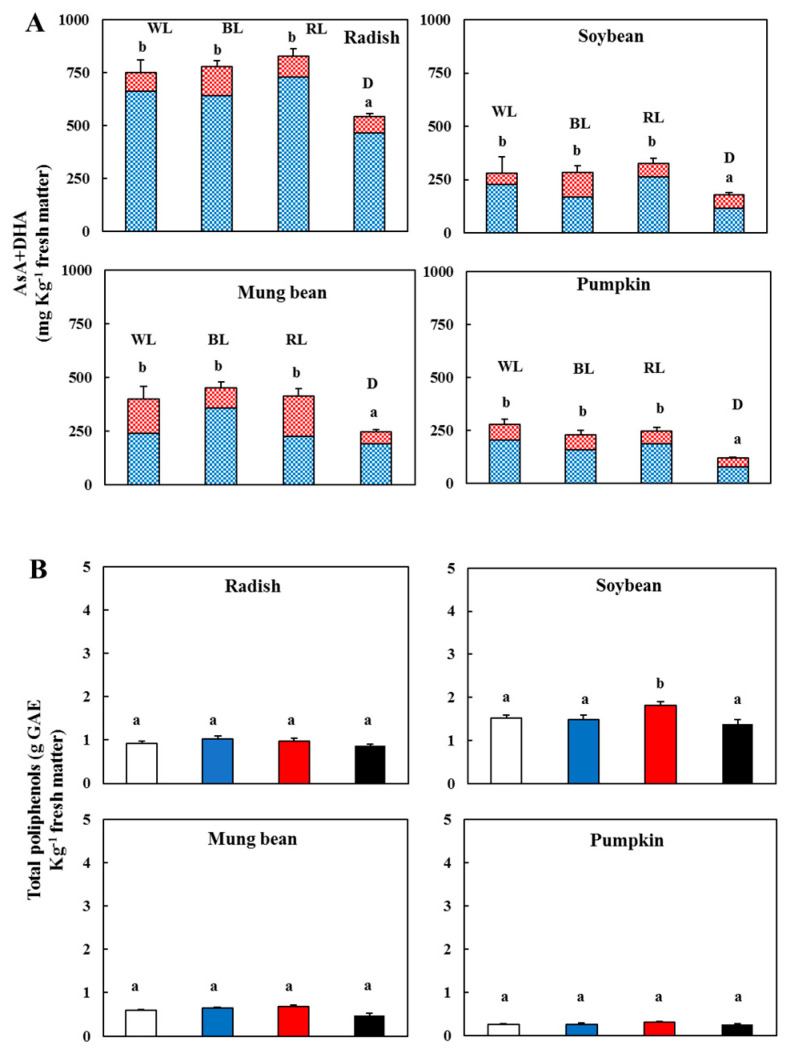
(**A**) Content of total ascorbate—l-ascorbic acid (AsA, 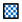) and l-dehydroascorbic acid (DHA, 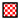)—In the sprouts 5 d after germination following various treatments. White light, WL; blue light, BL; red light, RL, and dark, D. (**B**) Total polyphenolic content in the sprouts 5 d after the germination of various treatments. White light (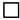); blue light (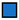); red light (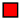), and dark (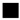). Values represent the mean of at least three replicates from four independent experiments. Identical letters over the columns indicate non-significant differences between treatments within each segment (Tukey’s HSD test, *p* < 0.05).

The quality and intensity of light are reported to be effective at regulating the AsA level in plants [13]. The lighting caused a significant (*p* < 0.05) increase in the ascorbate total content (AsA + DHA) in all the sprouts with respect to the dark (Figure 5A). Radishes contained the highest value, followed by mung beans and soybeans, while in pumpkins, the total content of ascorbate was lower in both the light and darkness. No significant difference was observed among the different light treatments for each type of sprout. Different dehydrated orthodox seeds did not contain AsA, although it is immediately produced after seed imbibition. It is reported that the AsA levels are low in dehydrated orthodox soybean seeds, and its strong increase during germination is due to the reactivation of its biosynthesis. In particular, the WL, BL and ultraviolet lights were more effective at activating L-galactono-γ-lactone dehydrogenase, the enzyme that converts the substrate L-Galactono-1,4-lactone into ascorbate [32]. In this study, we observed an increase in the AsA in the WL and BL treatments. However, the RL also induced the AsA increase in soybeans. In all the sprouts, the content of reduced ascorbate was higher than the oxidized form highlighting that the biosynthesis was higher than its degradation. Alternatively, the rapid increase in the AsA content and the activity of the enzymes of the ascorbate system is a fine strategy of defence in orthodox herbaceous plants seeds to counteract the high ROS level that occurs during germination [33]. Interestingly, the AsA content was the highest in the radishes whose cotyledons became active photosynthetic tissues.

The polyphenols are considered “scavengers” of free radical species and preserve the cell membranes from oxidative damage, thus benefitting human health. It is reported that various types of light influence the polyphenol content differently, and the responses were dependent on the species. The red LED increased the phenolic compound contents in common buckwheat sprouts but not in Tartary buckwheat sprouts [34]. Sweet basil grown under blue LED showed a lower total phenol content than that grown under white LED [35]. Qian and colleagues [36] reported that the vitamin C and total phenolic compounds of Chinese kale sprouts were not sensitive to the blue and red LED lights, respectively. However, our data indicated that when compared to the dark, the treatment with solely RL increased (*p* < 0.05) the phenolic content, while in the other sprouts, no difference between light and dark appeared (Figure 5B). Therefore, the light generally did not influence the biosynthesis of the total polyphenols in the various sprouts. Of note, soybeans, followed by radishes, had a higher content than mung beans and pumpkins.

### 3.4. Changes in the Anthocyanins, Chlorophylls and Carotenoids After Light and Dark Treatment

**Figure 6:**
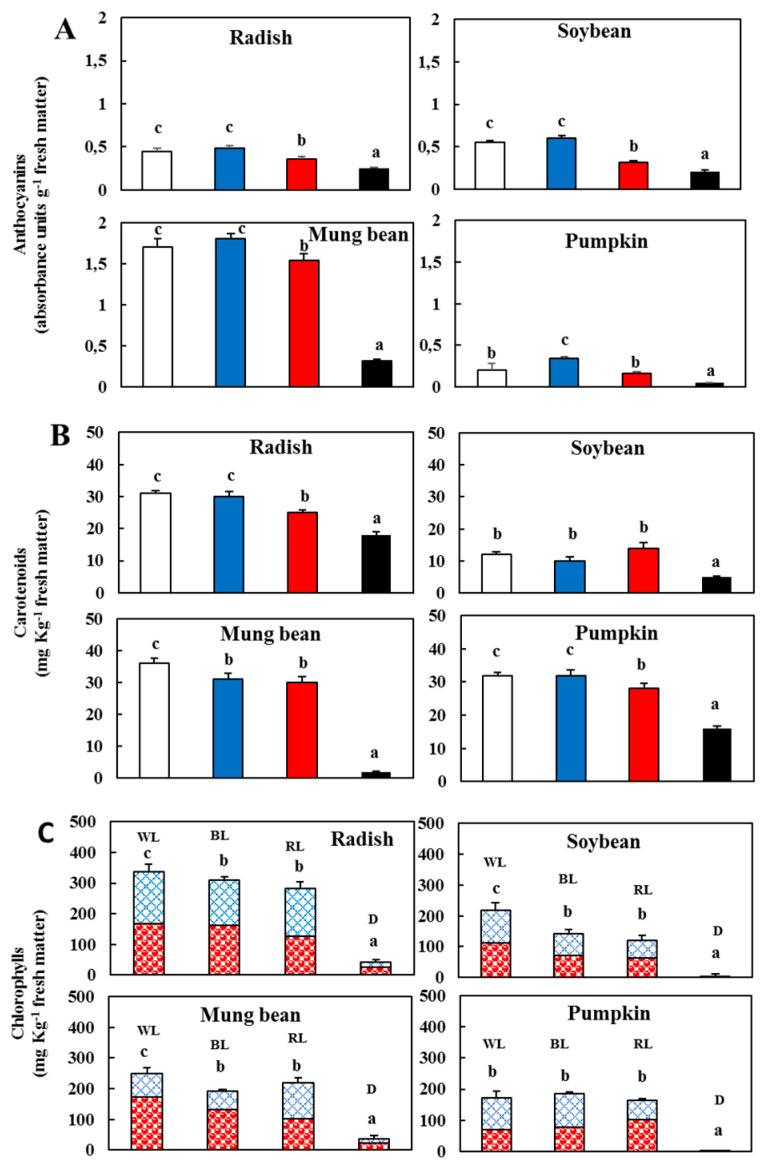
(**A**) Level of anthocyanins in the sprouts 5 d after germination following various treatments; (**B**) Carotenoid content in the sprouts 5 d after germination following various treatments. White light (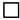); blue light (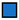); red light (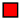), and dark (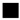). (**C**) Contents of chlorophyll a (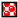) and b (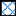) in the sprouts 5 d after germination following various treatments. White light, WL; blue light, BL; red light, RL, and dark, D. Values represent the means of at least three replicates from four independent experiments. Identical letters over the columns indicate non-significant differences between the treatments within each segment (Tukey’s HSD test, *p* < 0.05).

The various pigments examined in this work include the anthocyanins, whose synthesis is a process that is light-regulated and includes numerous steps starting from a phenylalanine precursor. Several plant species form anthocyanins in the light, while others do so in the dark, but their synthesis and content rapidly increases when exposed to light [37]. The inductive effect of light on anthocyanin synthesis was observable in all the sprouts (Figure 6A). The anthocyanin content was significantly (*p* < 0.05) higher in mung beans and soybeans in the various light conditions with the respect to the dark and had the highest increase in mung beans. The BL followed by the WL were more effective than the RL, while in pumpkins and radishes, a significant (*p* < 0.05) increase was observed only in the BL and WL compared to the dark. Indeed, the BL is reported to significantly induce the anthocyanin accumulation in *Arabidopsis* seedlings [16], Chinese bayberry fruit [38], apple fruit [39] and post-harvest strawberry fruit [40]. Alternatively, anthocyanin accumulation in young tissues, such as epicotyls and young leaves, is a common event in plants. As these organs lack morpho-anatomical complexity, the high presence of anthocyanins could prevent environmental stress, such as high lighting [41]. Indeed, anthocyanins that absorb wavelengths like those of chlorophyll b play an auxiliary role in photo-protecting the plant tissues.

The light also influences the chlorophylls and carotenoids. These pigments increased to the different spectral ranges. After lighting, the radish had a higher content of total chlorophyll (chl a + chl b; Figure 6C). Among the different light treatments, a higher value of chlorophyll was observed under the WL in all the sprouts, except for pumpkins. The highest increase in the carotenoids was in mung beans under the WL (Figure 6B). The lowest level of chlorophylls and carotenoids was observed in darkness for various sprouts, with the minimal value in mung beans. Interestingly, the increase in carotenoids, which are pigments that are components of the light-harvesting complex, has a protective role for the chlorophylls subjected to photo-oxidation reactions [42,43].

## 4. Conclusions

The frequent use of sprouts in vegetable diets is closely related to food safety and the nutritional benefit of their consumption. It is well known that both time and light influence seed germination. Consequently, different growth conditions might change the quality of the relative sprouts developed from the seed. In this study, after dark or light treatments to moderate irradiance (110 µmol m^−2^ s^−1^), the quality-related parameters were differentially influenced in the four types of sprouts analysed after 5 d of germination.

Compared to the darkness, the WL, RL and BL preserved the contents of vitamin C, carotenoids, chlorophylls, and anthocyanins in all the types of sprouts, highlighting how the use of a specific spectral wavelength increases the content of these antioxidant compounds. Their increase is useful, as they might be involved in the complex mechanism to prevent the oxidation of biological cell membranes. Interestingly, an increase in polyphenols was induced only in soybeans and only by RL. On the other hand, darkness preserved the dry matter, whereas the light decreased it.

Minimally processed sprouts may benefit from lighting during seed germination to improve their quality. The positive effects on vitamin C, carotenoids, chlorophylls, and anthocyanins in all sprouts, the increased soluble proteins and sugars respectively in soybean and pumpkin seeds, the increase of polyphenols in soybeans, together with low-cost lighting, and ease of implementation towards other vegetables greatly contribute to the light application in industrialized production. This will eventually increase the production of sprouts with higher nutritional/health value tailored for people with specific nutritional needs. Further studies will be needed to understand the molecular mechanism through which light modulates the synthesis of phytonutrients and changes the nutritional content of sprouted seeds, which can be beneficial to human health.
